# Validation of a novel smartphone-based photoplethysmographic method for ambulatory heart rhythm diagnostics: the SMARTBEATS study

**DOI:** 10.1093/europace/euae079

**Published:** 2024-03-27

**Authors:** Jonatan Fernstad, Emma Svennberg, Peter Åberg, Katrin Kemp Gudmundsdottir, Anders Jansson, Johan Engdahl

**Affiliations:** Karolinska Institutet, Department of Clinical Sciences, Danderyd University Hospital, Entrévägen 2, 182 88, Stockholm, Sweden; Department of Cardiology, Danderyd University Hospital, Entrévägen 2, 182 88, Stockholm, Sweden; Karolinska Institutet, Department of Medicine, Huddinge, Karolinska University Hospital, Stockholm, Sweden; Karolinska Institutet, Department of Clinical Sciences, Danderyd University Hospital, Entrévägen 2, 182 88, Stockholm, Sweden; Karolinska Institutet, Department of Clinical Sciences, Danderyd University Hospital, Entrévägen 2, 182 88, Stockholm, Sweden; Department of Clinical Physiology, Danderyd University Hospital, Stockholm, Sweden; Karolinska Institutet, Department of Clinical Sciences, Danderyd University Hospital, Entrévägen 2, 182 88, Stockholm, Sweden; Department of Cardiology, Danderyd University Hospital, Entrévägen 2, 182 88, Stockholm, Sweden

**Keywords:** Atrial fibrillation, Atrial flutter, Photoplethysmography, Telemonitoring, Smartphone, Mobile health

## Abstract

**Aims:**

In the current guidelines, smartphone photoplethysmography (PPG) is not recommended for diagnosis of atrial fibrillation (AF), without a confirmatory electrocardiogram (ECG) recording. Previous validation studies have been performed under supervision in healthcare settings, with limited generalizability of the results. We aim to investigate the diagnostic performance of a smartphone-PPG method in a real-world setting, with ambulatory unsupervised smartphone-PPG recordings, compared with simultaneous ECG recordings and including patients with atrial flutter (AFL).

**Methods and results:**

Unselected patients undergoing direct current cardioversion for treatment of AF or AFL were asked to perform 1-min heart rhythm recordings post-treatment, at least twice daily for 30 days at home, using an iPhone 7 smartphone running the CORAI Heart Monitor PPG application simultaneously with a single-lead ECG recording (KardiaMobile). Photoplethysmography and ECG recordings were read independently by two experienced readers. In total, 280 patients recorded 18 005 simultaneous PPG and ECG recordings. Sufficient quality for diagnosis was seen in 96.9% (PPG) vs. 95.1% (ECG) of the recordings (*P* < 0.001). Manual reading of the PPG recordings, compared with manually interpreted ECG recordings, had a sensitivity, specificity, and overall accuracy of 97.7%, 99.4%, and 98.9% with AFL recordings included and 99.0%, 99.7%, and 99.5%, respectively, with AFL recordings excluded.

**Conclusion:**

A novel smartphone-PPG method can be used by patients unsupervised at home to achieve accurate heart rhythm diagnostics of AF and AFL with very high sensitivity and specificity. This smartphone-PPG device can be used as an independent heart rhythm diagnostic device following cardioversion, without the requirement of confirmation with ECG.

## Graphical Abstract

**Graphical Abstract:**
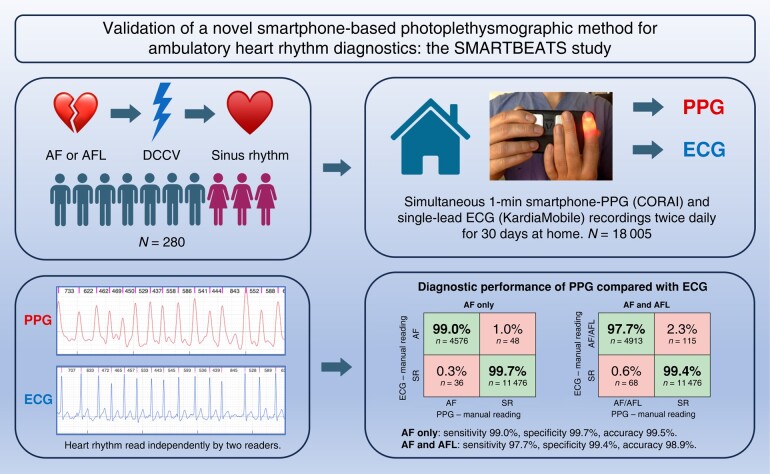

**What’s new?**

- First validation study of heart rhythm diagnostics using smartphone photoplethysmography (PPG) in an ambulatory real-world setting.
- Validated against simultaneous electrocardiogram recordings.
- Participants with atrial flutter included.
- The study showed excellent diagnostic performance for manual interpretation of PPG.

## Introduction

Atrial fibrillation (AF) is the most common cardiac arrhythmia, with a global prevalence of 60 million.^1^

Untreated AF increases the risk of all-cause death, ischaemic stroke, heart failure, and dementia.^2–5^ Atrial flutter (AFL) is a less prevalent cardiac arrhythmia, with similar symptoms, complications, and treatments as AF.^5^

European Society of Cardiology (ESC) guideline recommendation for AF diagnostics is a 12-lead electrocardiogram (ECG) (12L-ECG) recording, or a single-lead ECG (1L-ECG) tracing of at least 30 s, analysed manually by a physician with expertise in ECG rhythm interpretation.^5^ As smartphones are becoming ubiquitous worldwide, the availability of heart rhythm diagnostics and management of AF/AFL using smartphone photoplethysmography (PPG) is increasing. In the current ESC and American Heart Association guidelines, smartphone PPG is not being recommended for diagnosis of AF, without a confirmatory ECG recording.^6,7^

Different smartphone-PPG methods are not equivalent; in particular, aspects such as measurement technology, ease of use, resulting signal quality, and diagnostic performance differ.^8–10^

Concerns have been raised about the generalizability of the results of previous validation studies for various smartphone-PPG methods.^10^ Previous validation studies have been performed in a supervised healthcare setting, although most diagnostic use of smartphone PPG likely occurs in an unsupervised environment. Overall, the quality of the PPG recordings was often insufficient for diagnosis even though measurements were made under direct supervision.^11,12^ In addition, no previous study has validated smartphone PPG for AF detection compared with simultaneous ECG recordings. The use of smartphone PPG has been studied in ambulatory settings; however, these have been non-validation studies where the diagnostic performance of the PPG recordings was not evaluated in comparison with ECG recordings.^13–15^

This study was designed to investigate the diagnostic performance of a smartphone-PPG method in a real-world situation, using ambulatory unsupervised smartphone-PPG recordings compared with simultaneous 1L-ECG recordings, with heart rhythm diagnosis based on a single PPG recording, and including unselected direct current cardioversion (DCCV) patients with AFL and with AF.

## Methods

### Study design and participants

In this prospective validation study, participants were included at the Department of Cardiology, Danderyd University Hospital, Stockholm, Sweden. Unselected adult patients undergoing DCCV for treatment of persistent or recent-onset AF or AFL were included. Patients were included on weekdays and on days when research staff was available. Patients with a cardiac implantable electronic device were excluded.

### Smartphone-PPG device

Photoplethysmography is an optical method measuring variations in subcutaneous blood volume.^6^ The CORAI Heart Monitor PPG software application (Corai Medicinteknik AB, Stockholm, Sweden) is a Class IIb medical device that runs on the operating system of smartphones, such as the iOS (Apple Inc., Cupertino, CA, USA) and Android (Alphabet Inc., Mountain View, CA, USA) operating systems. CORAI uses the built-in sensors of the smartphone to record a high-resolution PPG measurement from the tip of the user’s finger positioned over the camera sensor (see Supplementary material online, *Figure S1*).

The resulting PPG recording contains physiological information, including current heart rhythm and heart rate.^6^ For each recording, a PPG report is automatically generated by the CORAI application, allowing for a heart rhythm diagnosis to be determined through manual reading (see Supplementary material online, *Figure S2*).

### Single-lead ECG device

KardiaMobile (AliveCor Inc., Mountain View, CA, USA) is a portable device that records a 1L-ECG when the user places fingers from opposing hands on the device’s two electrodes. KardiaMobile requires the presence of a smartphone that receives and processes the ECG during recording. KardiaMobile has been validated for the detection of several arrhythmias including AF.^16^ For each 1L-ECG recording, an ECG report is automatically generated, from which a heart rhythm diagnosis can be decided on by manual reading (see Supplementary material online, *Figure S2*).

### Simultaneous PPG and 1L-ECG recordings

**Figure 1:**
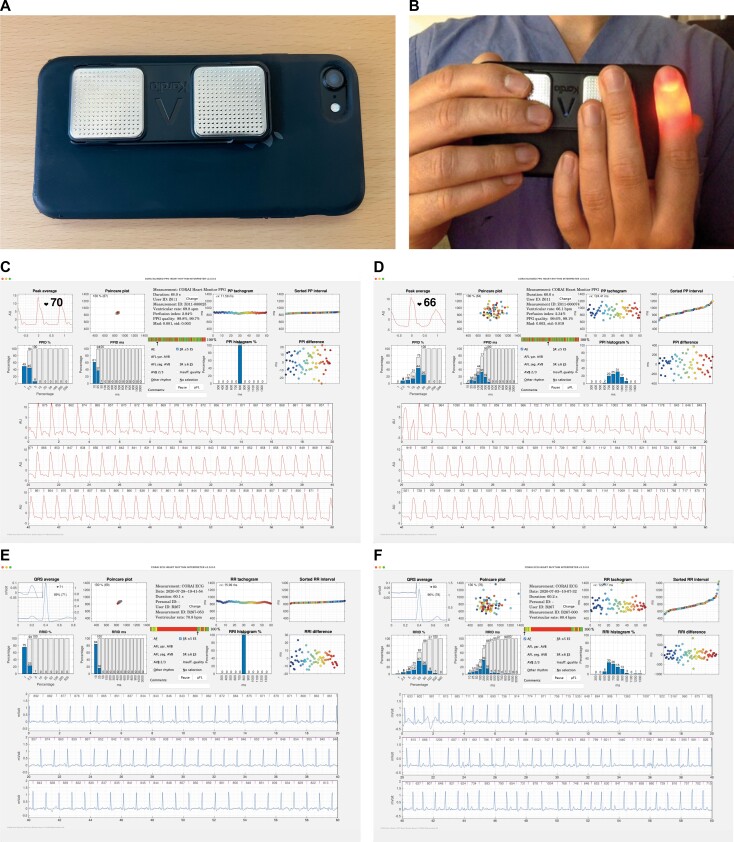
Simultaneous smartphone-PPG and single-lead ECG recordings with CORAI Heart Monitor and KardiaMobile. (*A*) The single-lead ECG device KardiaMobile was attached to the back of the smartphone case on an iPhone 7. (*B*) Shows the user handling and finger placements when performing a simultaneous smartphone-PPG recording with the CORAI Heart Monitor and single-lead ECG recording. The study participants recorded both types of recordings simultaneously by placing a fingertip over the camera lens of the smartphone and at the same time placing other fingers from both hands on the electrodes of the single-lead ECG device. (*C–F*) The user interface in the software application and the PPG and ECG reports used during manual reading for (*C–D*) the blinded smartphone-PPG recordings presented in random order and (*E–F*) the single-lead ECG recordings. The reader would select the heart rhythm diagnosis for the shown heart rhythm recording. For the recordings in *C* and *E*, the heart rhythm was sinus rhythm, and for *D* and *F*, it was atrial fibrillation.

The study participants were provided with an iPhone 7 (Apple Inc. Cupertino, CA, USA) smartphone to use for the study duration. The 1L-ECG device was attached to the back of the smartphone’s case. For each recorded smartphone PPG, a simultaneous 1L-ECG recording was made; see *Figure 1*. The study participants were given automatic real-time feedback of the user handling by the CORAI application during recordings to help preserve the signal quality of both PPG and ECG recordings. An update to the automatic real-time user feedback was made in the second half of the participant inclusion.

### Heart rhythm recordings

Patients eligible for DCCV were screened for participation on the day of treatment. After confirmation of AF or AFL on 12-lead ECG, and if no contraindications for DCCV were identified, patients were informed and asked about participation. Included patients were instructed to record simultaneous 1-min PPG and 1L-ECG measurements at least twice daily for 30 days following DCCV. The first simultaneous PPG and 1L-ECG recordings were made before the DCCV treatment. Patients not converted to sinus rhythm (SR) after DCCV, as recorded by 12-lead ECG, were excluded, and performed no further recordings. Recordings made after discharge were manually monitored daily by the investigators. Ambulatory recordings following discharge after DCCV were made unsupervised, i.e. without any assistance in device handling from the investigators. Patients with recurring recordings with heart rate lower than 40 or over 140 bpm had a telephone consultation with a physician.

### Manual reading of 1L-ECG and PPG recordings

The manual reading of 1L-ECG and PPG recordings used a customized software platform; see *Figure 1*. The set of heart rhythm diagnoses used is reported in Supplementary material online, table  *S1*. A minimum of 30 s of each 1-min recording was required to have sufficient signal quality to qualify for a heart rhythm diagnosis.

The 1L-ECG recordings were considered the gold standard and were independently analysed by two cardiology consultants (J.E. and K.K.G.) with experience in 1L-ECG reading. In the case of disagreement, a third cardiologist (E.S.) analysed the recording blinded to the heart rhythm diagnosis selected by the two initial readers. In case of disagreement between all three readers, heart rhythm diagnosis was decided on by consensus in a meeting. The 1L-ECG recordings for each pseudonymized patient were grouped together and presented chronologically.

The PPG recordings were independently analysed by two experienced readers (J.E. and J.F.). In the case of disagreement, a third experienced reader (A.J.) analysed the recording blinded to the heart rhythm diagnosis selected by the two initial readers. In case of disagreement between all three readers, heart rhythm diagnosis was decided on by consensus in a meeting. For the manual reading of the PPG recordings, each study participant was given a new random record ID eliminating the connection between PPG and 1L-ECG recordings. The PPG recordings for each patient were presented in random order eliminating any influence from the expected sequence of heart rhythm findings peri-DCCV.

Recordings lacking a heart rhythm diagnosis on either ECG or PPG, due to insufficient signal quality or for other reasons, were removed in the diagnostic performance calculations; see Supplementary material online, *Figure S3*.

### Statistics

Continuous variables, if normally distributed, were reported as mean with standard deviations, otherwise as median and interquartile ranges (IQRs) and dichotomous variables as proportions. The one-sample Kolmogorov–Smirnov test was used to test for normality. Diagnostic performance in terms of sensitivity, specificity, accuracy, and positive and negative predictive value (PPV and NPV) was calculated using 2 × 2 tables. Diagnostic performance was calculated separately with recordings on PPG or ECG read as AFL included and excluded.

Statistical significance for the diagnostic performance between subgroups of the study participants was calculated using a two-sided *χ*^2^ test. A *P*-value of <0.05 was considered significant.

The sample size was calculated for other outcomes studied in the project (NCT04300270); for the outcomes in the study related to diagnostic performance, the expected number of recordings for 280 study participants each contributing with an estimated 62 recordings was determined to be sufficient.^17^

### Ethics

The study was conducted according to the Declaration of Helsinki and was approved by the Regional Ethical Review Board, ref. no: 2018/1354–32, and by the Swedish Ethical Review Authority, ref. no: 2019–02150. All participants signed informed consent.

## Results

### Study population

**Figure 2:**
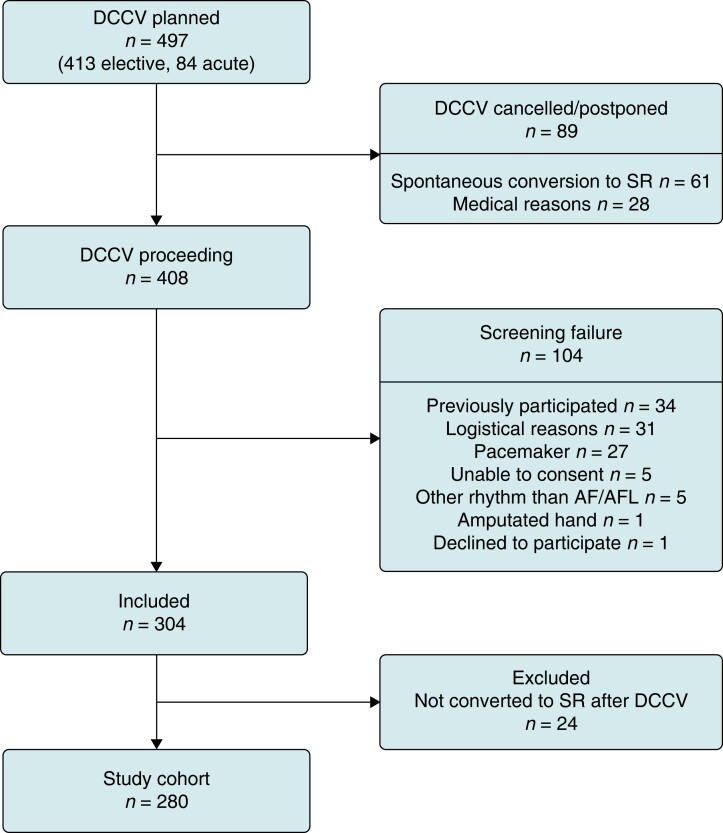
Study participant flow chart. Of the medical reasons for DCCV cancellation, the most common reason was non-compliance to anticoagulation therapy. The logistical reasons for screening failure included insufficient availability of study devices, DCCV procedure occurring before the patient could be asked for participation and inability to return the study device.

Between November 2018 and July 2020, 304 patients were included, of which 280 converted to SR after DCCV constituting the final study cohort (*Figure 2*). Of the 280 study participants, 214 (76.4%) had an elective DCCV procedure, and for 66 (23.6%), the procedure was performed within 48 h of AF/AFL initiation. Pre-DCCV 1L-ECG recordings were interpreted as AF in 82.1% (230/280), as AFL in 14.3% (40/280), and as having insufficient quality for diagnosis in 3.6% (10/280) of the participants. The baseline characteristics of the study population are shown in *Table 1*. Median age was 69.0 years and 31% (86/280) of the participants were female. The mean CHA_2_DS_2_-VASc score was 2.4 (median 2; IQR 2). The most reported EHRA score was III (52.5%).

**Table 1:** Baseline characteristics of the study population

Variable	*n* = 280
Age, median (IQR), years	69.0 (13.6)
Age, range, years	32.3–89.5
Female, *n* (%)	86 (30.7)
BMI kg/m2, median (IQR)	26.6 (5.1)
Pre-DCCV heart rhythm from 1L-ECG, *n* (%)	AF: 230 (82.1) AFL: 40 (14.3)
	Insufficient quality: 10 (3.6)
BP systolic, mmHg median (IQR)	130 (22)
BP diastolic, mmHg median (IQR)	85 (13)
Hyperlipidaemia, *n* (%)	63 (22.5)
Hypertension, *n* (%)	164 (58.6)
CHF, *n* (%)	53 (18.9)
Diabetes, *n* (%)	32 (11.4)
Stroke or TIA, *n* (%)	27 (9.6)
Vascular disease, *n* (%)	32 (11.4)
CHA_2_DS_2_-VASc score, mean, median, (IQR)	2.4, 2, (2)
EHRA score, proportion	I: 4.0%
	IIa: 9.7%
	IIb: 33.1%
	III: 52.5%
	IV: 0.7%
DCCV scheduled, *n*, (%)	214 (76.4)
DCCV within 48 h of AF/AFL initiation, *n*, (%)	66 (23.6)
First time DCCV, *n*, (%)	132 (47.1)
Number of previous DCCV, mean	2.78
Number of previous DCCV, range	0–35
Previous ablation procedure, *n* (%)	26 (9.3)
OAC treatment, *n* (%)	265 (94.6)
Warfarin, *n* (%)	18 (6.4)
DOAC, *n* (%)	247 (88.2%)
Mobile phone ownership, *n* (%)	279 (99.6)
Non-smartphone, *n* (%)	23 (8.2)
Smartphone, *n* (%)	256 (91.4)
iOS, *n* (%)	167 (65.2)
Android, *n* (%)	88 (34.4)
Windows Mobile, *n* (%)	1 (0.4)

BMI, body mass index; BP, blood pressure; CHF, congestive heart failure; DCCV, direct current cardioversion; DOAC, direct oral anticoagulants; IQR, interquartile range; OAC, oral anticoagulation; TIA, transient ischaemic attack.

In total, the study participants recorded 18 005 heart rhythm registrations using simultaneous PPG and ECG recordings. Rhythm recording adherence was good with a mean of 64.3 recordings out of 62 expected per participant (104%). In the study population, 91% reported smartphone ownership.

### Overall diagnostic performance

Manual reading of the PPG recordings diagnosed AF/AFL (sensitivity) in 97.7% [95% confidence interval (CI_95_): 97.3%–98.1%] and SR (specificity) in 99.4% (CI_95_: 99.3%–99.6%) of the recordings compared with manually interpreted ECG recordings, with an overall accuracy of 98.9% (CI_95_: 98.7%–99.1%). Positive predictive value and NPV were 98.6% (CI_95_: 98.3%–99.0%) and 99.0% (CI_95_: 98.8%–99.2%), respectively, including AFL recordings.

**Figure 3:**
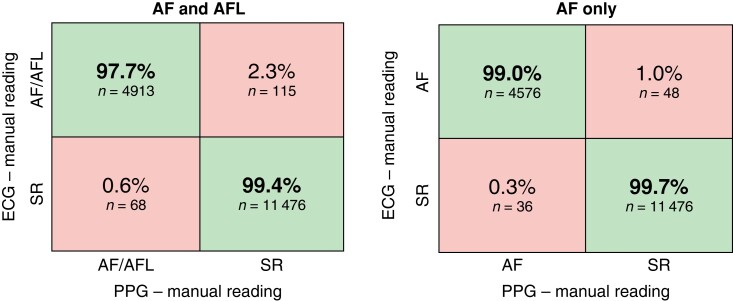
Overall diagnostic performance. This figure shows the diagnostic performance of manual reading of smartphone-PPG recordings with CORAI Heart Monitor compared with manual reading of the simultaneous single-lead ECG recordings with KardiaMobile, with AFL recordings included (left) and with AFL recordings excluded (right).

Results excluding recordings read as AFL on ECG or PPG diagnosed AF (sensitivity) in 99.0% (CI_95_: 98.7%–99.3%) and SR (specificity) in 99.7% (CI_95_: 99.6%–99.8%) of the recordings, with an overall accuracy of 99.5% (CI_95_: 99.4%–99.6%); see *Figure 3*. Positive predictive value and NPV were 99.2% (CI_95_: 99.0%–99.5%) and 99.6% (CI_95_: 99.5%–99.7%), respectively, excluding AFL recordings.

Excluding ECG or PPG recordings with AFL, 88.2% (247/280) of the study participants had complete agreement between all ECG and PPG recordings if dichotomized into normal vs. abnormal heart rhythm, and 95.7% (265/280) of the study participants showed at most one recording with disagreement. The corresponding results when AFL recordings were included were 80.4% (225/280) and 88.9% (249/280) of the study participants, respectively (see Supplementary material online, *Figure S4*).

### Manual reading of ECG recordings

Of the 1L-ECG recordings, 65.6% (11 820/18 005) were read as SR, 27.3% (4911/18 005) as AF, 2.0% (362/18 005) as AFL, and 4.9% (882/18 005) as having insufficient quality.

For 1.2% (223/18 005) of the 1L-ECG recordings, an inter-observer disagreement of the selected heart rhythm occurred (one of the ECG readers had selected SR, and the other reader had selected either AF or AFL). Of these inter-observer disagreements, the abnormal heart rhythm was AFL in 0.6% (107/18 005) and AF in 0.6% (116/18 005) of the 1L-ECG recordings. The proportion of AFL 1L-ECG recordings with inter-observer disagreement (29.6%, 107/362) was markedly higher than the respective proportion for AF (2.4%, 116/4911).

For ECG recordings with insufficient signal quality and for different variants of SR (see Supplementary material online, table  *S1*), the inter-observer disagreement was 3.7% (667/18 005) and 4.5% (802/18 005), respectively.

### Manual reading of PPG recordings

Of the PPG recordings, 68.1% (12 267/18 005) were read as SR, 26.7% (4808/18 005) as AF, 2.0% (367/18 005) as AFL, and 3.1% (561/18 005) as having insufficient quality.

For 2.5% (447/18 005) of the PPG recordings, an inter-observer disagreement between SR and AF/AFL occurred. Of these inter-observer disagreements, the abnormal heart rhythm was AFL in 1.3% (231/18 005) and AF in 1.2% (216/18 005) of the PPG recordings. Similarly, to the 1L-ECG recordings, the proportion of AFL PPG recordings with inter-observer disagreements compared with the total amount of PPG recordings read as AFL (62.9%, 231/367) was higher than the corresponding number for AF (4.5%, 216/4808).

For PPG recordings with insufficient signal quality and for different variants of SR (see Supplementary material online, table  *S1*), the inter-observer disagreement was 0.9% (159/18 005) and 1.7% (307/18 005), respectively.

### Quality of PPG and ECG recordings

**Figure 4:**
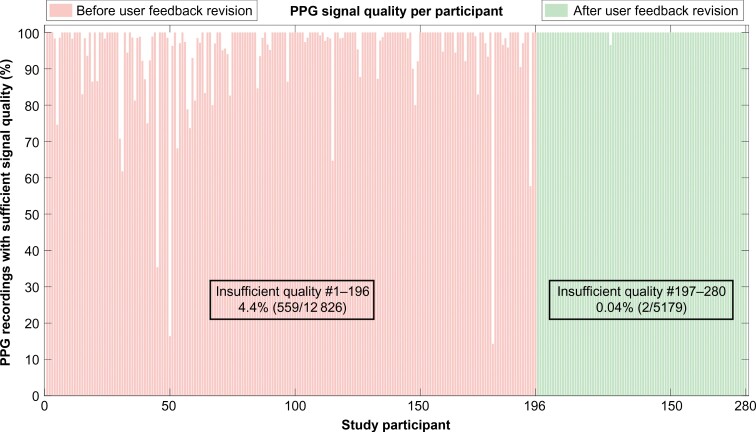
Signal quality of smartphone-PPG recordings. This figure shows the signal quality of the smartphone-PPG recordings for each of the study participants. The proportion of smartphone-PPG recordings with sufficient quality to make a heart rhythm diagnosis, as decided by manual reading, is shown on the *y*-axis. The study participants were given automatic real-time feedback of the user handling by the CORAI Heart Monitor application during the recordings to help preserve the signal quality of the recordings. A revision to this automatic real-time feedback improved the signal quality of the smartphone-PPG recordings further substantially. The revised feedback was used for the final 84 study participants, #197–280.

Of all the PPG recordings, 3.1% (561/18 005) had insufficient signal quality for diagnosis compared with 4.9% (882/18 005) of the ECG recordings (*P* < 0.001). For details on ECG signal quality per study participant, see Supplementary material online, *Figure S5*. Following a revision to the automatic real-time user feedback given during PPG recording for preserving signal quality, PPG signal quality improved (*Figure 4*). Of note, only 0.04% (2/5179) of the PPG recordings from the final 84 included patients had insufficient quality for diagnosis, compared with 4.4% (559/12 826) for the participants (*n* = 196) included prior to the real-time user feedback update. For details on rhythm recording classification, signal quality, and overlap between ECG and PPG, see Supplementary material online, *Figure S3*.

The diagnostic performance for the PPG recordings made by these final 84 participants was significantly higher with regard to sensitivity, accuracy, and NPV compared with the participants (*n* = 196) included prior to the real-time user feedback update, but was not significantly different for specificity and PPV (*Table 2*).

**Table 2:** Diagnostic performance before and after a revision to the automatic real-time user feedback that significantly increased the signal quality of the PPG recordings, with AFL recordings excluded

Diagnostic performance	Before revision	After revision	*P*
Participants (*n*)	196	84	—
Recordings	12 826	5179	—
PPG recordings with insufficient quality, *n* (%)	559 (4.4)	2 (0.04)	<0.0001
Sensitivity (%)	98.7 [98.4–99.1]	99.8 [99.5–100.0]	0.0030
Specificity (%)	99.7 [99.5–99.8]	99.7 [99.6–99.9]	0.5175
Accuracy (%)	99.4 [99.2–99.5]	99.7 [99.6–99.9]	0.0020
PPV (%)	99.3 [99.0–99.6]	99.0 [98.4–99.6]	0.3996
NPV (%)	99.4 [99.2–99.6]	99.9 [99.9–100.0]	<0.0001

Corresponding 95% confidence intervals are shown within brackets.

AFL, atrial flutter; NPV, negative predictive value; PPG, photoplethysmography; PPV, positive predictive value.

There was a significant difference (*P* = 0.004) in the proportions of PPG recordings with insufficient signal quality between smartphone owners (3.0%, 498/16 565, *n* = 256) and non-owners (4.4%, 63/1440, *n* = 24). For the final 84 participants, whose recordings were made with the automatic real-time user feedback update in place, there was no longer a significant difference between owners and non-owners (*P* = 0.59).

### Age and diagnostic performance

Diagnostic performance was compared between participants older and younger than the median age, without recordings read as AFL. The results were significantly different for sensitivity, accuracy, and NPV with slightly better performance for participants younger than the median age (*Table 3*).

**Table 3:** Diagnostic performance compared between the participants aged above and below median age, excluding AFL recordings

Age	Age < median	Age > median	*P*
Participants, *n*	140	140	—
Women, *n* (%)	25 (17.9)	61 (43.6)	—
Sensitivity (%)	99.4 [99.0–99.7]	98.7 [98.2–99.1]	0.0189
Specificity (%)	99.7 [99.5–99.8]	99.7 [99.6–99.8]	0.7359
Accuracy (%)	99.6 [99.5–99.7]	99.4 [99.2–99.5]	0.0351
PPV (%)	99.0 [98.5–99.4]	99.4 [99.1–99.7]	0.0953
NPV (%)	99.8 [99.7–99.9]	99.3 [99.1–99.6]	0.0001

Corresponding 95% confidence intervals are shown within brackets.

AFL, atrial flutter; NPV, negative predictive value; PPV, positive predictive value.

### Ambulatory recordings

The study participants initially performed a few supervised recordings (median 3, mean 3.0) before and directly after their DCCV treatment and then continued with unsupervised ambulatory recordings in their home environment (95.4%, 17 169/18 005). Diagnostic performance was compared between the subset of recordings performed in the supervised and the ambulatory settings, without recordings read as AFL, and was significantly higher for specificity, accuracy, and NPV for the ambulatory recordings (*Table 4*).

**Table 4:** Diagnostic performance compared between recordings made supervised and recordings made ambulatory, excluding AFL recordings

Setting	Supervised		Ambulatory	*P*
Participants, *n*		280		—
Recordings, *n* (%)	836 (4.6)		17 169 (95.4)	—
Sensitivity (%)	97.8 [95.9–99.7]		99.0 [98.7–99.3]	0.0812
Specificity (%)	99.2 [98.4–100.0]		99.7 [99.6–99.8]	0.0441
Accuracy (%)	98.8 [98.0–99.6]		99.5 [99.4–99.6]	0.0059
PPV (%)	98.3 [96.6–100.0]		99.3 [99.0–99.5]	0.0883
NPV (%)	99.0 [98.1–99.9]		99.6 [99.5–99.7]	0.0370

Corresponding 95% confidence intervals are shown within brackets.

AFL, atrial flutter; NPV, negative predictive value; PPV, positive predictive value.

## Discussion

In this study, we report a high diagnostic performance for smartphone-PPG heart rhythm recordings as compared with 1L-ECG in an ambulatory elderly population. Despite using an ambulatory population, the proportion of recordings with insufficient signal quality was low compared with previous validation studies.

This is the first smartphone-PPG validation study performed in an ambulatory, real-world setting. Previous validation studies of smartphone PPG for AF detection have been performed in supervised healthcare settings, which is in discordance with the likely user scenario.^10^ Performing validation in an ambulatory setting should provide more realistic data from the intended setting and intended use of the device. Validation of a heart rhythm device in the ambulatory setting includes several challenges compared with in-office settings; for instance, supervision from healthcare professionals is not readily available and adherence could be difficult to achieve.^18^ Even though previous studies all were in supervised healthcare settings, the results in this unsupervised study show great diagnostic performance in comparison.^10^

### Ambulatory recordings

Previous studies have reported high adherence to intermittent ambulatory ECG recordings when performed daily during a limited period of a few weeks,^19,20^ whereas studies using more long-term infrequent monitoring with handheld devices have reported more limited compliance.^18^ In this study, we observed good adherence to heart rhythm recordings twice daily for a period of 30 days. The diagnostic performance was significantly higher for several metrics for the unsupervised recordings compared with the few supervised recordings made initially by each study participant. This is likely due to several factors, including that the supervised recordings were made initially when the study participants were new to these types of recordings. The supervised recordings were also made in conjunction with the cardioversion procedure, which might have affected the patients. The unsupervised ambulatory recordings were in contrast made in a calmer, non-healthcare setting, which may have been a contributing factor to these differences in diagnostic performance.

Some diagnostic metrics showed better results in the younger half of the study population. Frailty in the older population, including impaired motor function and tremor, and possibly having a lower digital literacy are factors that could have contributed to this difference.

### Simultaneous ECG recordings

When investigating the diagnostic performance of a diagnostic device, performing simultaneous recordings with the reference device constitutes a considerable advantage^21^ and specifically for a heart rhythm device since the recordings from the two devices would be identical beat for beat, thus eliminating the risk of any changes in heart rhythm affecting the validation. Not using simultaneous recordings has been reported as a limitation in previous validation studies.^11,12^

### Manual reading

In clinical practice, it has been shown that automatic algorithm-based methods are more readily accepted by physicians if the method is easy to comprehend and considered familiar and transparent.^22^ For heart rhythm diagnostics by smartphone PPG to achieve broader acceptance, it seems important that healthcare professionals can understand and interpret the PPG recordings by manual reading,^13^ which ideally should be a process as straightforward for a trained reader as manual interpretation of an ECG. The high diagnostic accuracy seen by experienced readers in this study suggests that manual reading of smartphone PPG for heart rhythm diagnostics is a skill that can be learnt by healthcare professionals.

### Study design

Previous validation studies of smartphone-PPG methods for AF detection have been found to generally be of varying quality, with concerns of a high risk of bias particularly related to patient selection and exclusion of data from final analysis.^10^ A need for validation studies to be more real-world-like has been identified.^10,12^

In several previous studies, the heart rhythm diagnosis has been decided from the majority outcome of multiple consecutive PPG recordings, in conflict with real-world usage where only a single recording is used.^12,23,24^

A previous validation study performed smartphone-PPG recordings with five times longer recording duration than what the method being validated uses in real-world situations, but then also presented results for shorter durations by selecting PPG fragments having the highest signal quality from within the longer recordings. Presenting such results for shorter duration fragments does probably not give an optimal representation of real-world diagnostic performance.^11^

Multiple validation studies have pre-selected the study participants by scanning their medical records to find the most suitable patients. Using such non-probabilistic sampling method for patient inclusion in a validation study will increase the risk of lower generalizability of the method being validated.^11,12,25,26^

### Atrial flutter

All previous smartphone-PPG heart rhythm validation studies have excluded patients with AFL. Atrial flutter is often a challenging diagnosis using 1L-ECG.^27,28^ To mimic a real-world setting and to aid in the generalizability of the results, we chose to include patients with AFL in this study. There was an over-representation of AFL in inter-reader disagreements, and diagnostic performance was slightly reduced when AFL recordings were included. Atrial fibrillation recordings had better diagnostic performance than AFL recordings, likely since there is less risk of confusing AF with other rhythms, compared with AFL, which can be confused with SR, SR with frequent extra-systoles, sinus tachycardia, and supraventricular tachycardia.

### PPG signal quality

In previous validation studies for smartphone-PPG methods reporting PPG signal quality, 17%^12^ and 32%^11^ of the recordings had insufficient quality for diagnosis, despite being recorded in an office setting under supervision. In this study, the number of total PPG recordings having insufficient quality for diagnosis was low (3.1%) compared with previous validation studies, and the proportion of PPG recordings with insufficient quality improved further to 0.04% for the final 84 included participants included after a revision to the automatic real-time user feedback, compared with 4.4% for the 196 participants included prior.

The signal quality of the PPG recordings had an effect on the diagnostic performance. Improvement in signal quality led to significantly better diagnostic performance in several metrics, emphasizing the importance of smartphone-PPG methods having an effective automatic system for real-time user feedback, or a similar process, that can help with improving the signal quality of the recordings.

### Strengths and limitations

This study has several strengths, including investigating the diagnostic performance of a smartphone-PPG method in a real-world situation, using ambulatory unsupervised smartphone-PPG recordings, compared with simultaneous ECG recordings, investigating diagnostic performance for manual reading of smartphone PPG, with heart rhythm diagnosis based on a single PPG recording and including unselected DCCV patients with AFL as well as with AF.

Another strength of the study is the large number of smartphone-PPG recordings, markedly higher than what has been included in any previous validation study, which assists with the accuracy of the findings.

The PPG recordings exhibited high signal quality, resulting in a high proportion of PPG recordings having sufficient quality for diagnosis, and likely made the manual reading process easier and contributing to the high diagnostic performance achieved.

Another strength is that the smartphone-PPG recordings for each study participant were presented to the readers in a random order, eliminating any influence from the expected sequence of heart rhythm findings peri-DCCV.

This study has several limitations, among them using a 1L-ECG device as the reference method. The 1L-ECG has limitations compared with 12L-ECG for arrhythmia diagnosis.^27^ The 1L-ECG reference device has been validated against 12L-ECG for the diagnosis of AF.^16^

As simultaneous smartphone-PPG and 1L-ECG recordings were performed, the user experience and handling were not identical to the standard situation, where only a PPG or an ECG is recorded, which might have resulted in lower quality recordings of both PPG and 1L-ECG recordings compared with non-simultaneous recordings.

Having only a 1L-ECG available could make a diagnosis of AFL more difficult.^27,28^ Thus, for 1L-ECG, there is a higher risk for misclassifications between AFL and other rhythms, as compared with multi-lead ECG.

These validation results should be considered very robust for AF, but, in comparison, less so for AFL since the proportion of AF recordings compared with AFL recordings was high. However, in absolute numbers, the included AFL recordings (*n* = 362) in this study are in line with, and in many cases exceeding, the number of included recordings with AF in previous validation studies.^10^

The study population constituted an elderly population but was limited to patients undergoing DCCV; thus, AF/AFL patients considered unsuitable for DCCV were not participating.

## Conclusion

A novel smartphone-PPG method can be used by patients unsupervised at home to achieve accurate heart rhythm diagnostics of AF and AFL with very high sensitivity and specificity. This smartphone-PPG device can be used as an independent heart rhythm diagnostic device following cardioversion, without the requirement of confirmation with ECG.

## Supplementary Material

euae079_Supplementary_Data

## Data Availability

The data underlying this article will be shared on reasonable request to the corresponding author.
