# Supplementary material for: Validation of a novel smartphone-based photoplethysmographic method for ambulatory heart rhythm diagnostics: the SMARTBEATS study
Source: Europace. 2024 Mar 27;26(4):euae079. doi: 10.1093/europace/euae079 (PMC11023506; doi:10.1093/europace/euae079)
Supplement: euae079_Supplementary_Data [file euae079_supplementary_data.docx]

# Supplementary data

| **A**  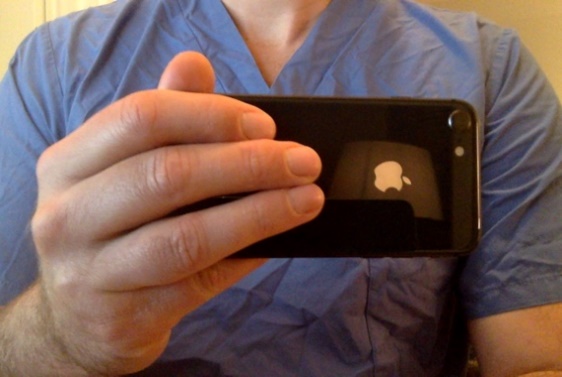  **B**  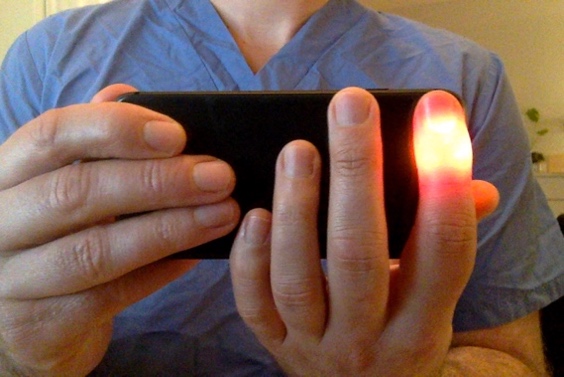 | **C**  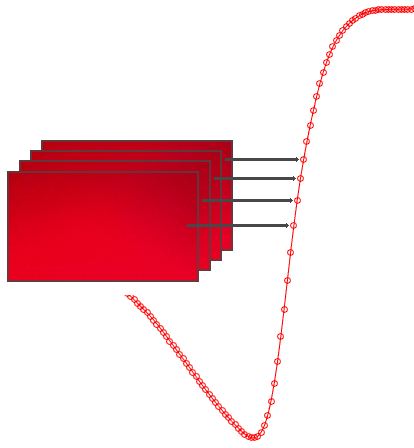 |
| --- | --- |
| **Supplementary figure S1** Smartphone-PPG recording with CORAI Heart Monitor. (*A*, *B*) Shows user handling and finger placements when performing a standard smartphone-PPG recording with the CORAI Heart Monitor by placing the tip of a finger over the camera lens of a smartphone. (*C*) Illustrates how the CORAI Heart Monitor application, running on the smartphone, analyzes each image frame from the camera and reduces it to a single sample point in a resulting PPG curve. | |

| **A** 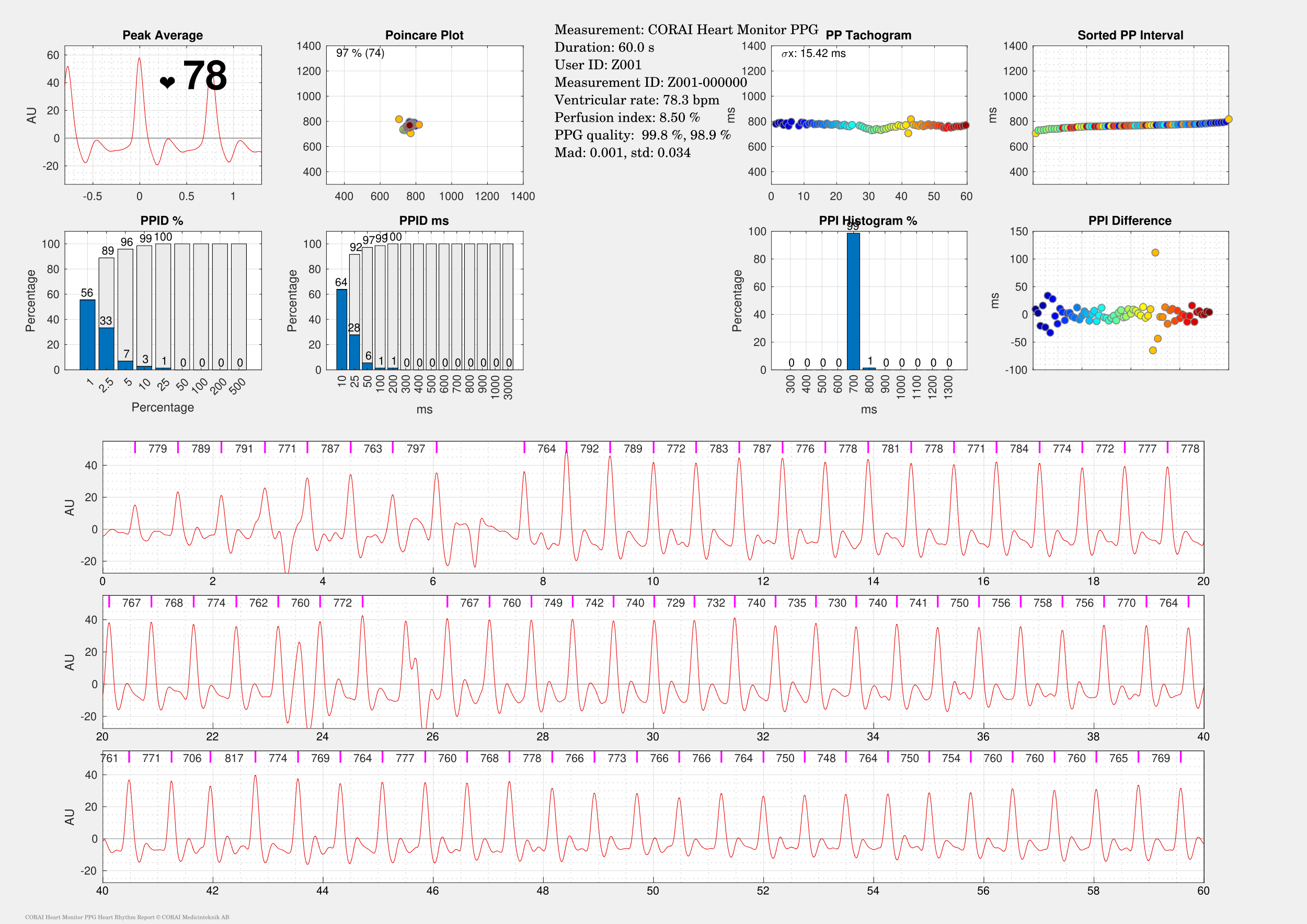 | **B**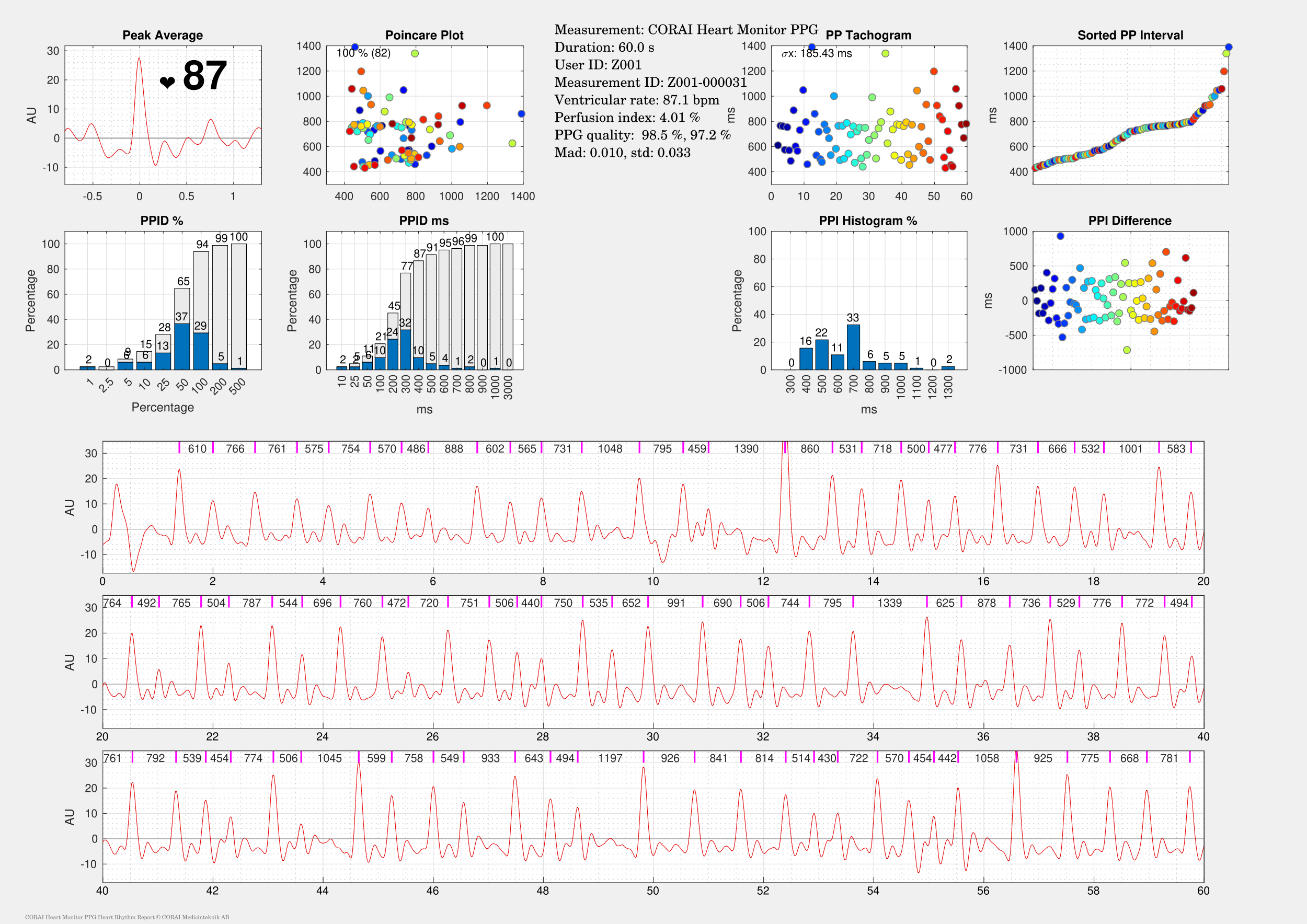 |
| --- | --- |
| **C**  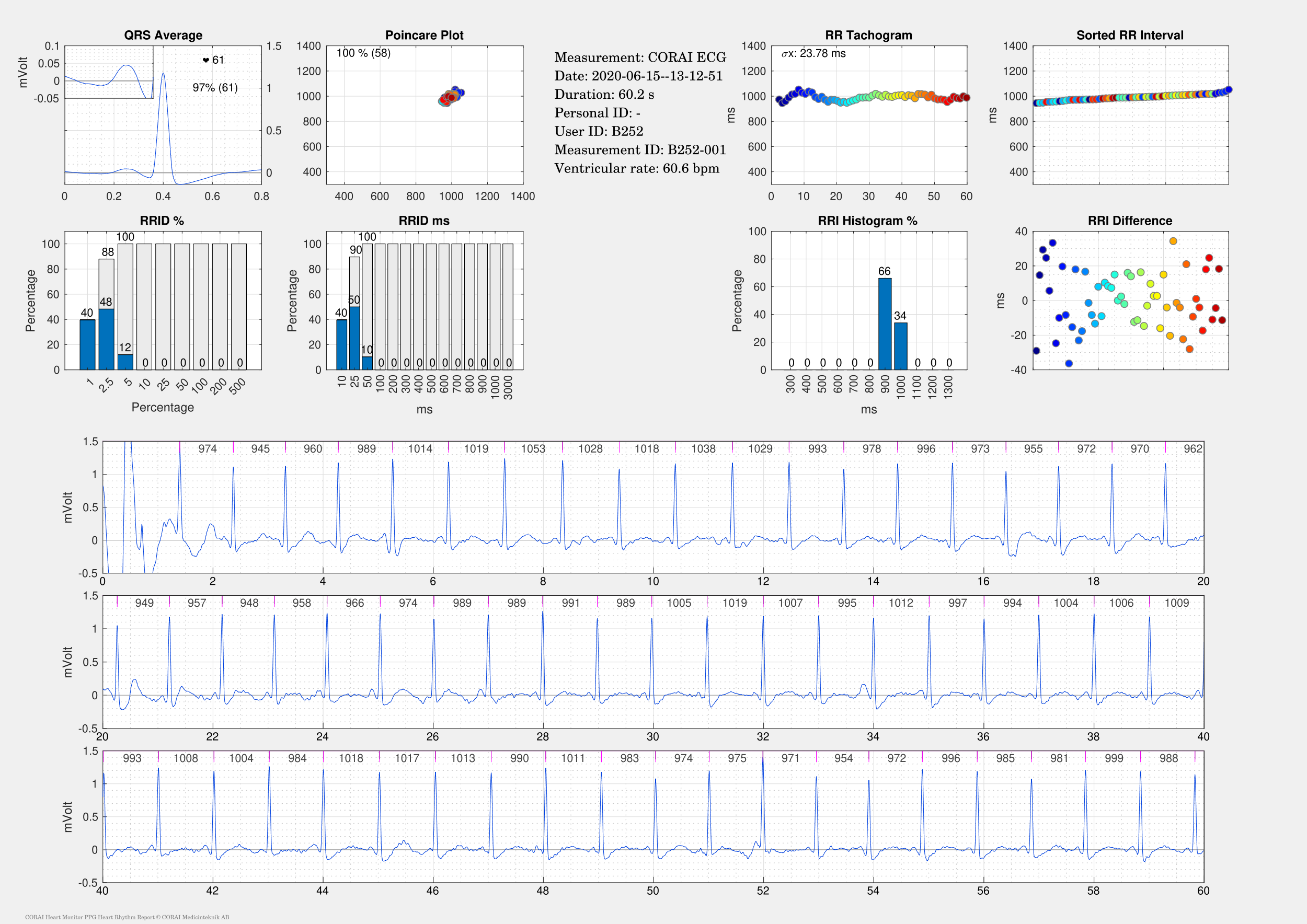 | **D**  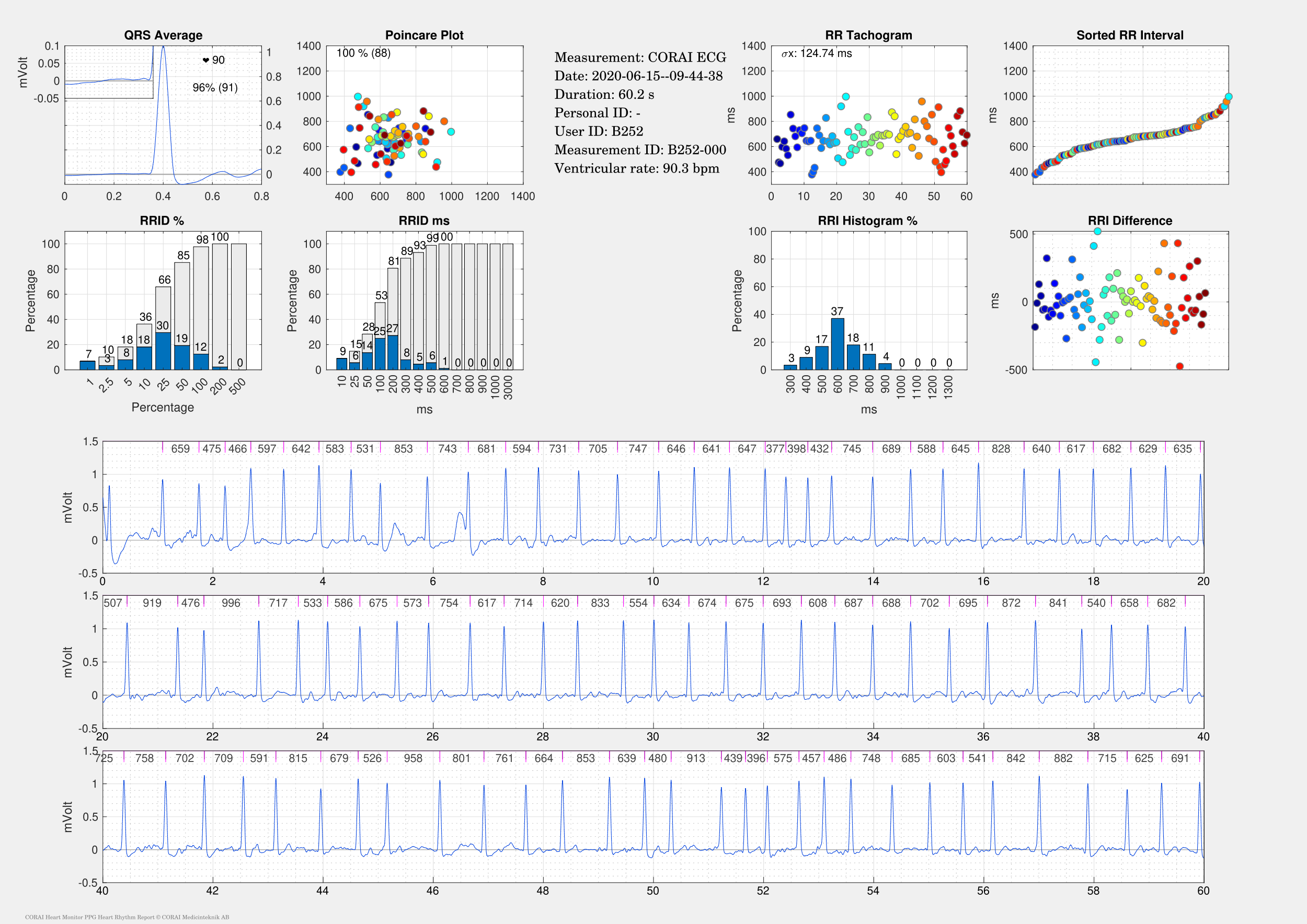 |
| **E** 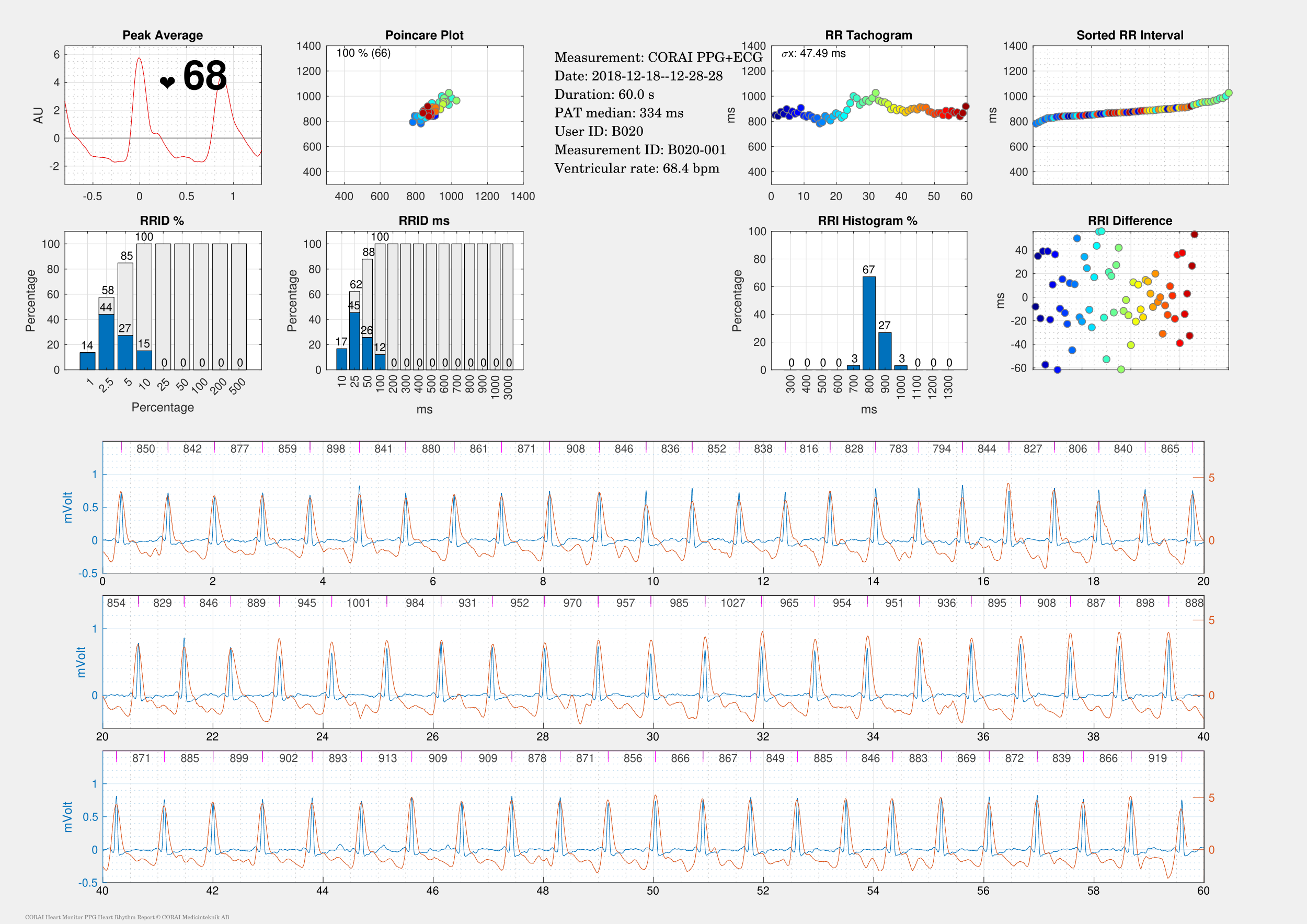 | **F** 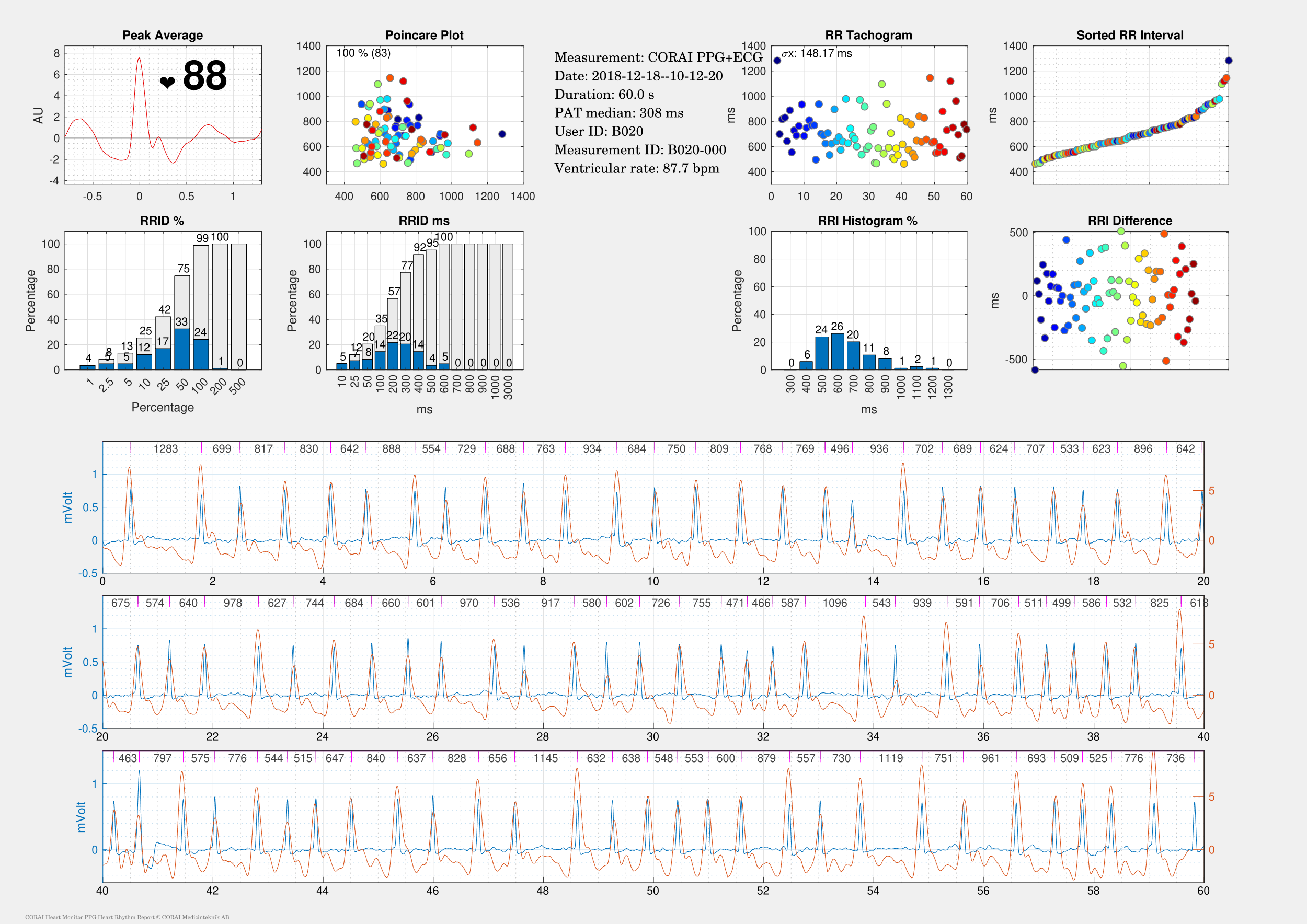 |
| **Supplementary figure S2** CORAI Heart Rhythm report. (*A-B*) Example of resulting CORAI Heart Rhythm PPG reports from smartphone-PPG recordings using the CORAI Heart Monitor. (*C*-*D*) CORAI Heart Rhythm ECG report from a single-lead ECG recording with the KardiaMobile ECG device. (*E*-*F*) Simultaneous smartphone-PPG recordings with the CORAI Heart Monitor and single-lead ECG recording shown overlaid in the same report. The heart rhythm in (*A*, *C*, *E*) was sinus rhythm, while (*B*, *D*, *F*) shows recordings for the corresponding patient when the heart rhythm was atrial fibrillation. | |

| 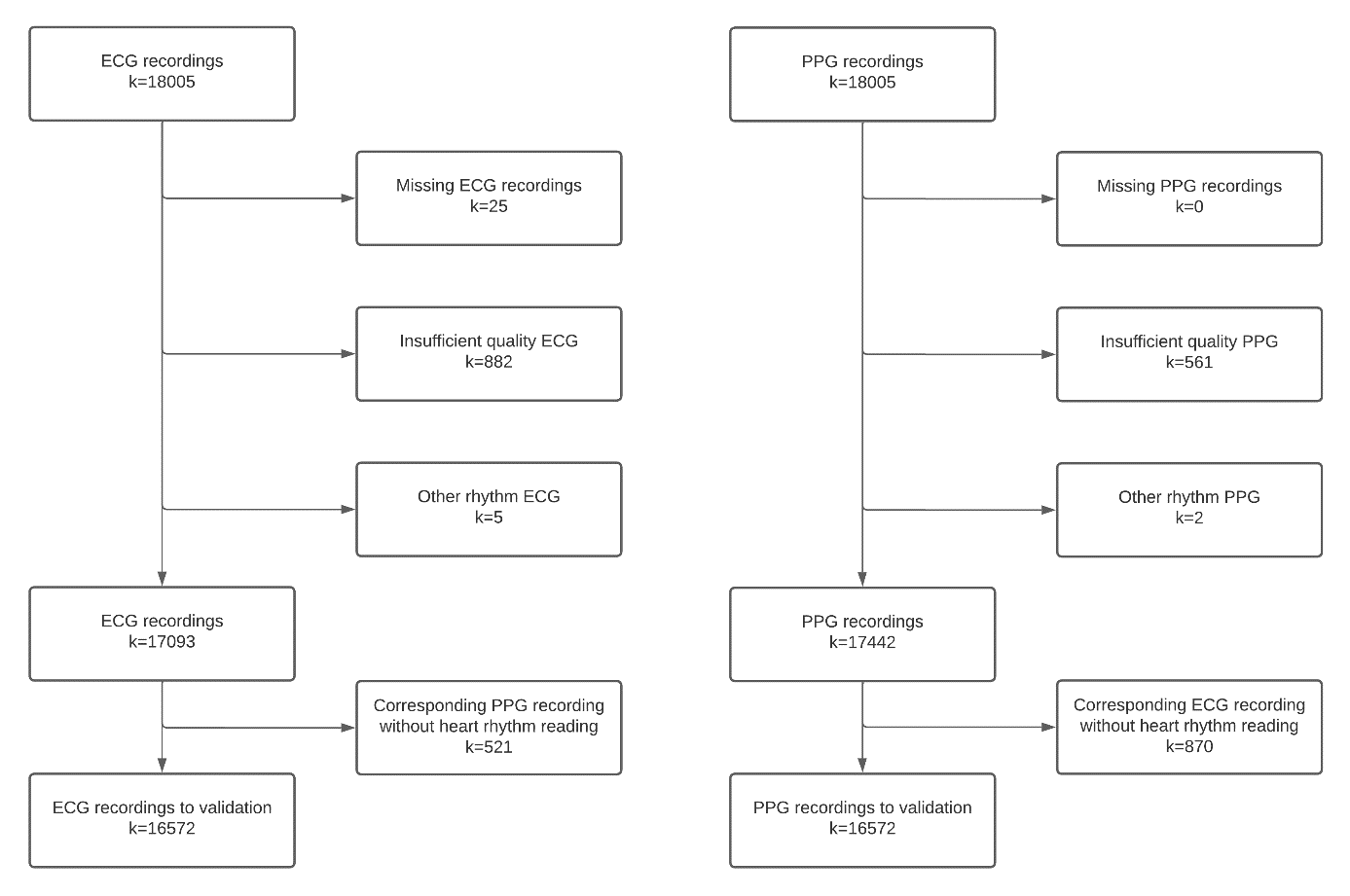 |
| --- |
| **Supplementary figure S3** PPG and ECG recordings to validation**.** To calculate diagnostic performance, heart rhythm diagnoses for both the smartphone-PPG recording and the simultaneous single-lead ECG recording must be present. Reasons for lack of diagnosis include missing recordings, insufficient quality, and other heart rhythms. “Other rhythm” was mostly selected for recordings having a heart rhythm change mid-recording. |

| **A**  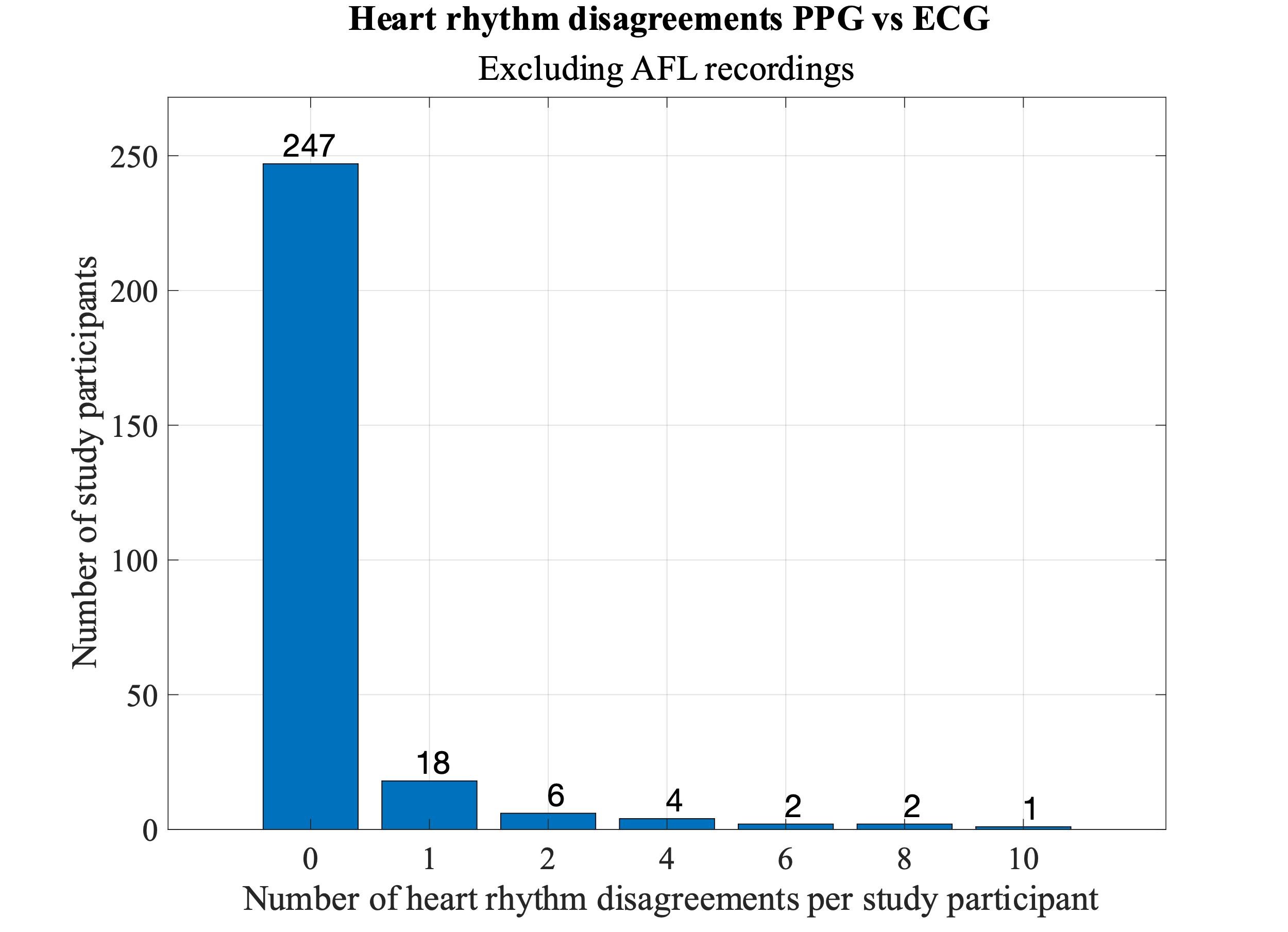 |
| --- |
| **B**  **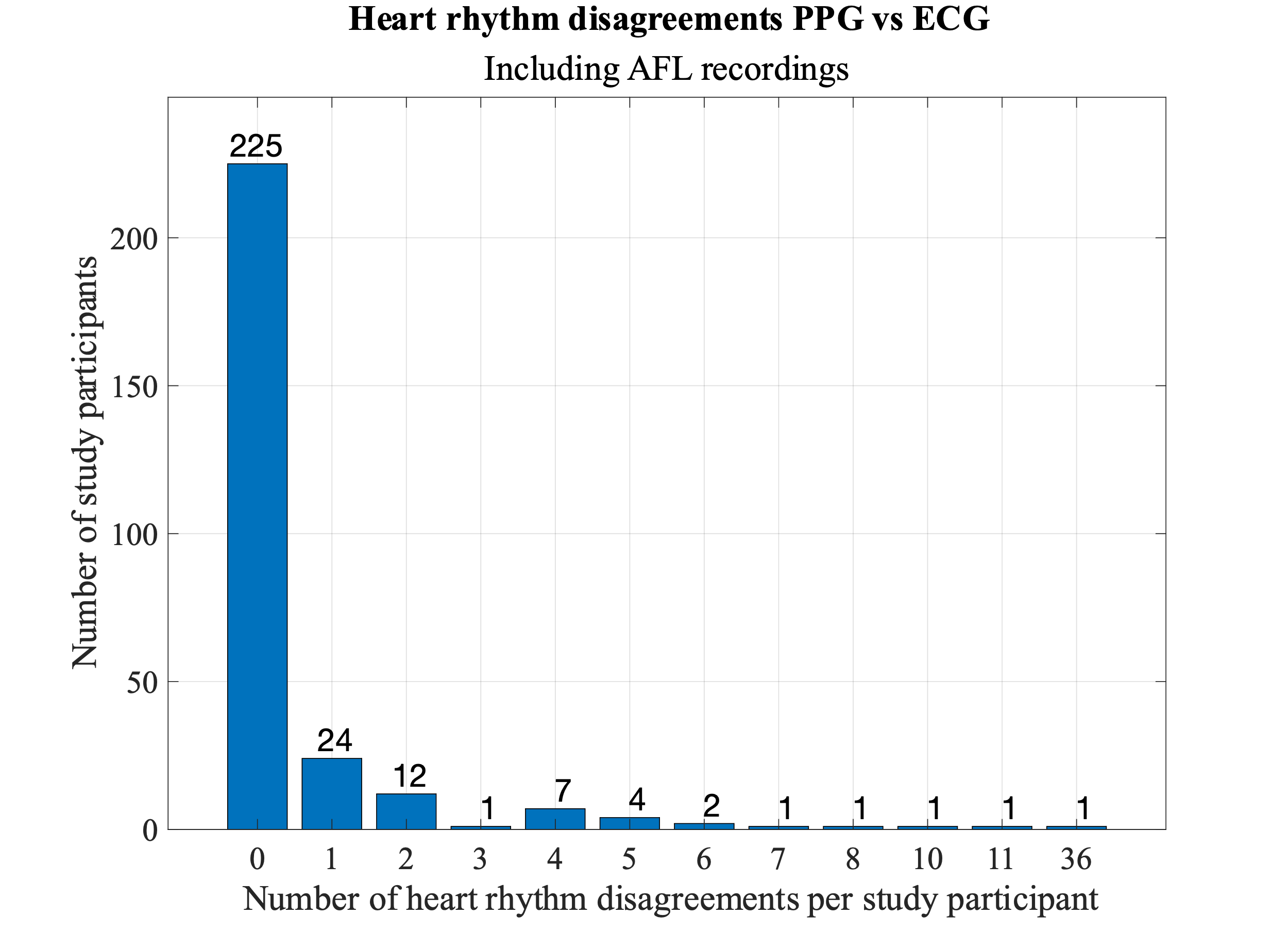** |
| **Supplementary figure S4** Diagnostic accuracy per study participant. Diagnostic accuracy for manual reading of heart rhythm from smartphone-PPG recordings using CORAI Heart Monitor per study participant (*A*) excluding AFL recordings and (*B*) including AFL recordings. The number of study participants with the same number of disagreements in the read heart rhythm between smartphone-PPG and 1L-ECG are displayed together. Excluding AFL recordings, 247 out of the 280 (88.2%) study participants showed no disagreement at all, and 265 (95.7%) of the study participants showed at most one disagreement. |
| 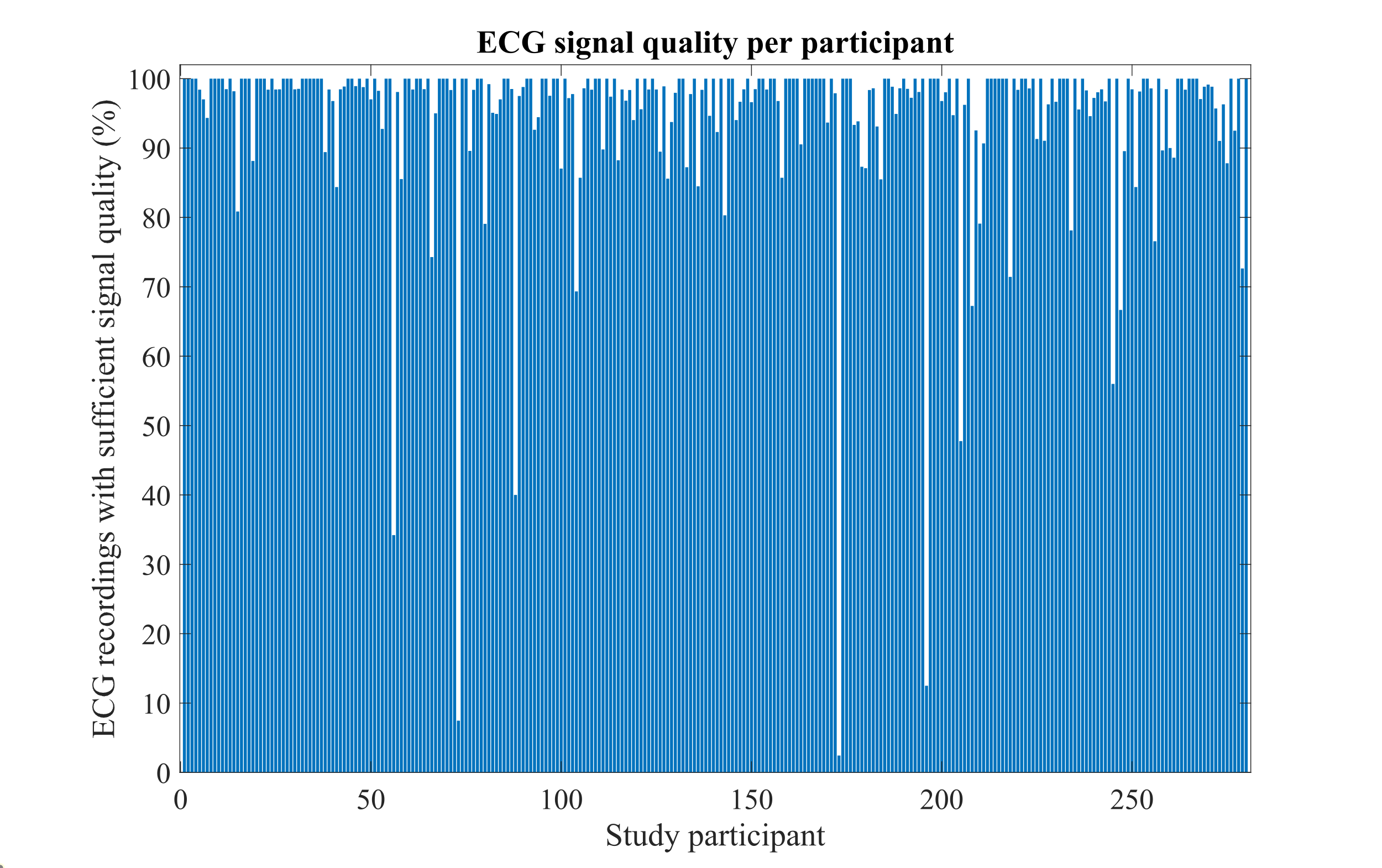 |
| **Supplementary figure S5** Signal quality of single-lead ECG recordings. Signal quality of the single-lead ECG recordings with KardiaMobile for each of the study participants. The proportion of single-lead ECG recordings with sufficient quality to make a heart rhythm diagnosis, as decided by manual reading, is shown on the y-axis. The study participants received automatic real-time feedback on the user handling from the CORAI Heart Monitor application during the recordings to aid in preserving the signal quality of the single-lead ECG recordings. |

| Supplementary table S1 The set of heart rhythm categories available to choose from during manual reading of smartphone-PPG and ECG recordings | |
| --- | --- |
| Atrial fibrillation | Sinus rhythm, with ≤ 5 ectopic beats |
| Atrial flutter, with regular AV conduction | Sinus rhythm, with ≥ 6 ectopic beats |
| Atrial flutter, with variable AV conduction | Insufficient signal quality |
| AV block II/III | Other rhythm |
